# Breathing Room: Industrial Zoning and Asthma Incidence Using School District Health Records in the City of Santa Ana, California

**DOI:** 10.3390/ijerph19084820

**Published:** 2022-04-15

**Authors:** Kelton Mock, Anton M. Palma, Jun Wu, John Billimek, Kim D. Lu

**Affiliations:** 1 Program in Medical Education for the Latino Community, University of California Irvine School of Medicine, Irvine, CA 92617, USA; kamock@hs.uci.edu; 2 Institute for Clinical & Translational Science, University of California Irvine, Irvine, CA 92697, USA; palmaa@hs.uci.edu; 3 Department of Environmental and Occupational Health, Program in Public Health, University of California Irvine Susan and Henry Samueli College of Health Sciences, Irvine, CA 92617, USA; junwu@hs.uci.edu; 4 Program in Medical Education for the Latino Community, Health Policy Research Institute, Department of Family Medicine, University of California Irvine, Irvine, CA 92617, USA; jbillime@hs.uci.edu; 5 Department of Pediatrics, University of California Irvine School of Medicine, Irvine, CA 92617, USA

**Keywords:** asthma prevalence, obesity, children, pediatric, industrial zone, industry, zoning, Latinx, Latino, Hispanic

## Abstract

Background: Traffic and industrial emissions are associated with increased pediatric asthma morbidity. However, few studies have examined the influence of city industrial zoning on pediatric asthma outcomes among minoritized communities with limited access to air monitoring. Methods: In this cross-sectional analysis of 39,974 school-aged students in Santa Ana, CA, we investigated the effect of proximity to areas zoned for industrial use on pediatric asthma prevalence, physical fitness, school attendance, and standardized test scores. Results: The study population was 80.6% Hispanic, with 88.2% qualifying for free/reduced lunch. Compared to students living more than 1 km away from industrial zones, those living within 0.5 km had greater odds of having asthma (adjusted OR 1.21, 95% CI 1.09 to 1.34, *p* < 0.001). Among children with asthma, those living between 0.5–1.0 km had greater odds of being overweight or obese (aOR 1.47, 95% CI 1.00, 2.15, *p* = 0.047). Industrial zone proximity was not significantly associated with worse fitness and academic outcomes for students with asthma. Conclusion: These findings suggest that industrial zone proximity is associated with increased pediatric asthma in a predominantly Latino community in Southern California.

## 1. Introduction

Industrial zoning decisions, which are vital in shaping the visual and economic landscapes of cities, can have powerful impacts on human health, often in patterns that disproportionately harm minoritized groups [1]. Among the many different environmental disturbances caused by industrial activities, air pollution is especially insidious, and has been associated with severe health effects, including increased asthma prevalence and morbidity among children [2,3,4]. A growing body of evidence for pollution-related asthma morbidity comes from literature on traffic-related air pollution (TRAP) showing the significant effects of nitrogen oxides and particulate matter on the incidence of pediatric asthma [5,6,7,8,9,10,11,12,13]. One important finding emerging from TRAP studies is the delineation of minimum distances, ranging from 0.075 km to 0.5 km, within which home proximity to major roads and highways constitute a significant risk factor for asthma. Increased asthma morbidity, in turn, can have serious consequences on children’s health and quality of life, including lower physical fitness and increased school absenteeism [14,15,16,17,18,19,20,21,22,23]. Studies of academic outcomes have consistently demonstrated significant differences in absenteeism among children with and without asthma, and, in some cases, have demonstrated worsening standardized test scores as a result [19,20,21,22,23]. Children with asthma have also been found to have difficulties engaging in physical activity, and obesity and asthma are frequently observed as co-morbid conditions in pediatric populations [14,15,16,17,18].

Studying the effects of air pollution directly is limited by the availability of monitoring data, as air monitors are costly to maintain; thus, extant air monitoring data is often interpolated from sparse networks of stationary air monitors [24]. Indirect, distance–gradient approaches are used to characterize the health effects of proximity to an area of suspected harmful environmental exposures, which is valuable as an initial tool in describing environmental risk and identifying potential exposures of interest [25]. This is supported by studies of industrial zoning exposure on pediatric asthma that have found increased asthma prevalence, asthma symptoms, exacerbations, and healthcare encounters in relation to specific types of pollutants [26,27,28]. However, there remains a knowledge gap regarding the relationship between broader patterns of industrial zoning and pediatric asthma morbidity [29,30,31,32].

The city of Santa Ana, CA, is 78.2% Hispanic/Latino (among whom 70.1% report Mexican ancestry), has a lifetime pediatric asthma incidence of 13.8% versus the national average of around 11.4–11.6%, and contains 17 census tracts labeled “disadvantaged communities” by the State of California [33,34,35,36,37]. At present, real-time air pollutant levels for Santa Ana are estimated from readings of a single monitoring station over 10 miles away, in the city of Anaheim [38]. Although long-standing community concerns about children’s health and environmental exposures have led to the recent acquisition of resources to develop its community air monitoring network, this network, based on low-cost sensors, only measures fine particulate matter concentrations and cannot capture toxic emissions from industrial facilities. Questions and concerns remain about whether home and school distance to areas zoned for industrial use might be associated with pediatric health outcomes, even in the absence of neighborhood-level pollutant data [39].

The aim of this study was to examine relationships between industrial zone proximity and asthma prevalence, as well as the effects of industrial zone proximity on academic and health outcomes among children with asthma, using student records from the Santa Ana Unified School District (SAUSD).

## 2. Materials and Methods

The study was conducted according to the guidelines of the Declaration of Helsinki and approved by the Institutional Review Board of UC Irvine (protocol #2019-5117, approval 10 June 2019) and the SAUSD Research and Evaluation Department.

### 2.1. Sample Selection

**Figure 1:**
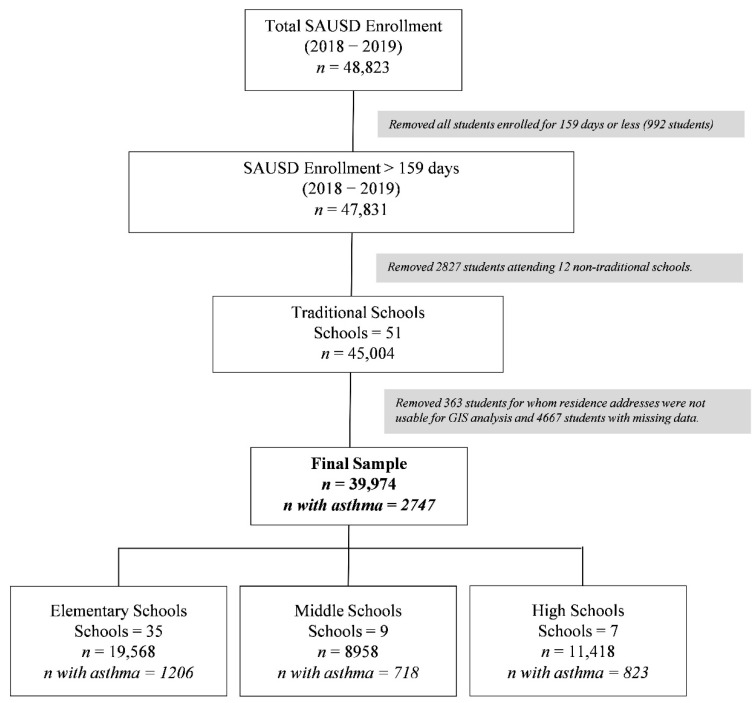
Study sample selection. The final sample included 39,974 students at 51 schools.

K-12 student data were obtained on all SAUSD enrolled students for the 2018–2019 school year, including demographic information, student medical records, physical fitness test data, and standardized academic testing data. Of the 48,823 total student records from 63 schools, we excluded 2827 students who attended 12 non-traditional schools (head-start programs and remedial programs for adults) and 992 students who were enrolled for 159 days or fewer to ensure sufficient enrollment time to ascertain outcomes. Home and school addresses were geocoded using ArcGIS Pro TM (Redlands, CA). In total, 363 students for whom only a PO box home address was available (*n* = 239), whose residential address fell outside of Orange County (*n* = 87), or whose geocoding was of low accuracy (i.e., geocoding matching score < 85; *n* = 19), or otherwise not available (*n* = 18), were considered ineligible for geocoding home address and were excluded from the analysis [40,41]. We further excluded 4667 students due to missing data on key variables for analysis, yielding a final sample size of 39,974 (Figure 1).

### 2.2. Outcome Variables

Asthma diagnosis and medical or health appointment-related absences were extracted from SAUSD records for the 2018–2019 school year. Per SAUSD nursing staff, diagnoses are updated in the student’s health record at regular intervals, which may vary across schools. We defined asthma diagnosis as having an asthma diagnosis on school records, and/or evidence of albuterol or inhaler use at school, and/or evidence of an asthma-related event at school in their school medical record. Data on the number and type of student absences were imported from SAUSD records. Any type of absence linked to health needs, including absences coded as “health appointments,” “illness”, or “medical absence” was defined as a medically related absence.

Fitness outcomes were obtained from FITNESSGRAM physical fitness tests, which are administered to students in grade levels 5, 7, and 9 [42]. Of the 11,180 students in these grades, 10,996 (98%) had a documented physical fitness test score. Of the different tests assessed in FITNESSGRAM, the aerobic capacity assessment was used as an outcome measure given the known relationship between severe asthma and diminished aerobic capacity [43,44]. Students were classified as having a healthy or unhealthy aerobic fitness score using established FITNESSGRAM scoring cutoffs.

Height and weight were measured for all students and were used to calculate BMI percentile relative to the US pediatric population and adjusted for sex and age [45]. For this analysis, children with BMI equal to or greater than the 85th national percentile were classified as overweight/obese.

The statewide Smarter Balanced (SB) assessments for mathematics and English/language arts were available for *n* = 21,938 and 21,931 students, respectively, across grades 3–8 and 11. Details of the SB assessment are available through the California Department of Education. Briefly, the assessments are designed to test students’ knowledge levels based on statewide grade-level standards. Students were classified as having achieved or failed to meet their grade-level standard of knowledge [46].

### 2.3. Exposure Variables

Industrial zones were demarcated according to the City of Santa Ana Zoning Map, corresponding to areas zoned for either heavy or light industrial use [47]. Freeways included interstate and state highways. The shortest home or school distance in kilometers to the nearest freeway or boundary of an industrial zone polygon was then calculated using ArcGIS Pro. We combined home and school distances to calculate weighted composite distance variables by incorporating the proportion of time students stay at home or school using the formula: 0.21 × distance from school + 0.79 × distance from home, which corresponds roughly to 7 h × 5 days per week over the total number of hours in a week. We then categorized the distance variables roughly into tertiles, with the farthest group being treated as the reference category (distance to industrial zone: closest < 0.5 km, middle 0.5–1.0 km, farthest > 1.0 km; distance to freeway: closest < 1.5 km, middle 1.5–3.0 km, farthest > 3.0 km).

### 2.4. Analysis

Sample characteristics were examined using means and standard deviations (SD) for continuous measures, and frequencies and proportions for categorical measures and compared between students with asthma vs. without asthma using independent samples *t*-tests and chi-squared tests, respectively. To test the hypothesized relationships between industrial zone or freeway proximity and asthma, fitness, and academic outcomes, generalized linear regression models were fit on each outcome adjusted for demographic covariates, with logit link for binary outcomes (asthma diagnosis, overweight/obese vs. normal weight, failed aerobic fitness test, failed SB math test, and failed SB English test) and identity link for continuous outcomes (number of absences). Fitness and academic outcomes analyses were completed among students with asthma only. Effect estimates were expressed as adjusted odds ratios (aORs) or mean differences and 95% confidence intervals (CIs) for each outcome for each tertile closer to nearest industrial zone or freeway. Adjusted models included sex, age, self-reported Hispanic ethnicity, parents’ education (at least one parent completed high school), qualifying for free or reduced lunch, and zip code. Results were considered significant at a level of *p* < 0.05. All analyses were conducted using R statistical software, v4.1.1 (R Core Team (2020), Vienna, Austria.

## 3. Results

### 3.1. Sample Characteristics

The study sample included 39,974 students, among whom the mean age was 12.2 ± 3.6 years, 50.5% were male, 80.6% identified as Hispanic, 88.2% qualified for free or reduced lunch, and 47.7% reported that neither parent had completed high school (Table 1). Fifty percent of students were overweight or obese and the mean number of days absent was 5 ± 7.3 days. The prevalence of asthma diagnosis was 6.9% (*n* = 2747). Students with asthma were more likely than students without asthma to be male, overweight, and fail their aerobic test but not academic tests. Mean composite distances were 0.9 km to the nearest industrial zone and 2.2 km to the nearest freeway.

### 3.2. Asthma

**Figure 2:**
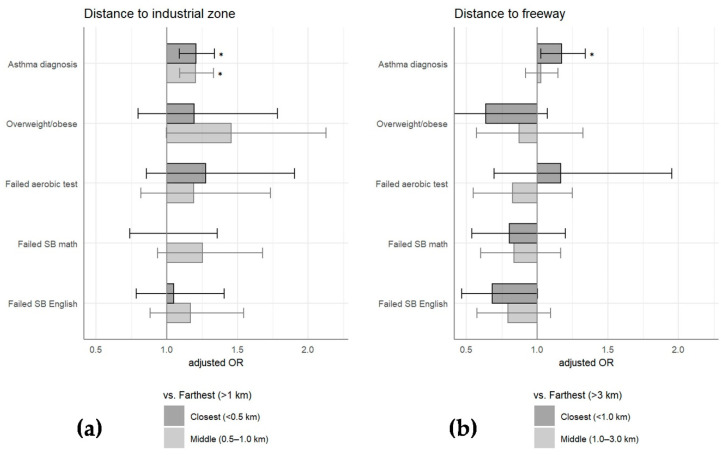
Regression models for asthma and academic outcomes by distance to industrial zone and freeways. Adjusted odds ratios with 95% CI shown among closest distance group vs. farthest distance group (darker grey) and middle distance group vs. farthest distance group (lighter grey) for (**a**) industrial zones and (**b**) freeways. * with *p* < 0.05.

The odds of having an asthma diagnosis were 21% greater among the middle distance group vs. farthest distance group to industrial zone (aOR = 1.21, 95% CI 1.10–1.34, *p* < 0.001) and also 21% greater among the closest vs. farthest distance group to industrial zone (aOR = 1.21, 95% CI 1.09–1.34, *p* < 0.001; see Table 2 and Figure 2). Proximity to the nearest freeway was associated with 18% higher odds of asthma diagnosis when comparing the closest vs. farthest distance groups only (middle vs. farthest group: aOR = 1.03, 95% CI 0.92, 1.16, *p* = 0.555; closest vs. farthest group: aOR = 1.18, 95% CI 1.03, 1.35, *p* = 0.014; Table 2 and Figure 2).

### 3.3. Obesity and Fitness Outcomes among Children with Asthma

Among students with asthma, an elevated risk of being overweight or obese was observed for those with moderate proximity (0.5–1.0 km) to industrial zones only (aOR 1.47, 95% CI 1.00 to 2.15, *p* = 0.047; Table 2 and Figure 2). The distance to the nearest freeway was not associated with a risk of obesity. Failure on the aerobic FITNESSGRAM test was not significantly associated with distance to industrial zones or the nearest freeway.

### 3.4. Academic Outcomes and Absences among Children with Asthma

A majority of students failed to achieve adequate scores (i.e., were scored as “needs improvement” or below) on the Smarter Balanced math and English tests. No significant associations were found between distance to industrial zone or freeway and failure of either Smarter Balanced math or English tests, which may be partly due to the high prevalence of failure. Likewise, distance to industrial zones and nearest freeway were not associated with total or health-related absences (see Table 2 and Figure 2).

## 4. Discussion

This study examined the effects of residential and school proximity to industrial zones and freeways on several health and academic outcomes among a sample of 39,974 K-12 students in the majority poor and Latinx city of Santa Ana, California, using readily available school records. As hypothesized, we found that increased proximity to industrial zones was associated with higher asthma prevalence when controlling for socioeconomic factors. The magnitude of this association was uniform for those with close and moderate proximity to industrial zones, defined as <1.0 km. Among children with asthma, industrial zone proximity was also associated with a greater likelihood of being overweight or obese, although this finding was only significant for those with moderate proximity. However, we observed no significant association between industrial zone proximity and school absences or standardized test scores. Proximity to freeways was associated with increased asthma prevalence among those in the closest category, but not associated with any of the other health or academic outcomes.

This study was conceived in collaboration with residents of Santa Ana, CA and SAUSD stakeholders in response to longstanding concerns about a possible relationship between industrial zoning and pediatric asthma incidence. A major strength of this study was its cost-effective use of available school district records, which contain standardized academic and fitness records as well as school absences and health records in relation to industrial zone/freeway proximities. Using the simple distance–gradient method of exposure analysis, our industrial zone findings support the results of prior studies that showed the deleterious effects of myriad industrial activities on asthma outcomes [25,26,27,28,48]. In particular, our analysis found increased asthma risk for children within 1 km of industrial zones, which is consistent with prior risk zones identified for proximity to chipboard industries (<2 km) and industrial parks (<5 km) [28,48]. Others using distance–gradient modeling have also found decreased lung function, increased ED visit rates, increased and hospitalizations related to proximity to industrial sources (petrochemical plants, wood processing industries), which was not available in our dataset [26,28]. We also observed an increased risk of overweight/obesity with industrial zone proximity, which corroborates previous findings suggesting worsening metabolic outcomes for children exposed to industrial pollution [49].

In contrast to prior studies linking traffic-related air pollution with worse asthma health, we found weak evidence for the effects of proximity to freeways on asthma risk, and no significant evidence for a link between freeway exposure and obesity, aerobic fitness, or academic outcomes among children with asthma. This is potentially explained by our tertile cut-points for freeway distance (<1.5 km, 1.5–3.0 km, >3.0 km); these are unlikely to adequately capture freeway pollutants, which have been shown to disperse to background levels at a distance of 200–2000 m, depending on meteorological conditions [50,51]. These cut-points were chosen based on the distribution of distance to freeways in this sample, but sensitivity analyses using alternative cut-points did not substantially alter the results (data not shown). Taken together, industrial zone and freeway exposure results suggest a patterning of health risk around industrial zoning and further study of possible factors/mechanisms, including increased air pollution, industry-associated traffic and/or noise, pollution from intermediate “trunk roads”, and other household and neighborhood-level factors that may affect asthma risk [52].

Consistent with previous studies, we found that our students with asthma had higher mean numbers of days of total and medically related absences compared to their peers [19,20,21,22,23]. However, we did not find any significant differences in academic outcomes between children with and without asthma or related to proximity to industrial zones among the students with asthma; however, there was a high rate of failure on both the standardized math and English tests among the student population. Overall, the data are mixed for academic outcomes related to asthma; some studies report no differences in academic outcomes despite differences in absenteeism among students with asthma, while other have demonstrated associations between asthma and worsening standardized test outcomes [19,20,21,22,23]. Differences in asthma diagnosis or severity as well as the demographic and socioeconomic characteristics of the various student populations may be contributing factors.

Among children with asthma, our finding of a possible increased risk of obesity for children at medium and close distances to areas of industrial zoning supports previous studies suggesting that exposure to unmeasured, industry-associated chemical obesogens, may play an important role in obesity disparities [53]. The combination of both asthma and obesity may increase susceptibility to pollutant exposure [54]. Additionally, children living closest to industrial areas may face other neighborhood risk factors, such as limited health care access, decreased access to green spaces, lower neighborhood walkability, and diminished availability of healthy food options [55,56]. Though we controlled for parents’ education and neighborhood effects with zip code, we did not have direct measures of socioeconomic status. Likewise, 10% of students were dropped from this analysis due to missing data; thus, it is possible that some selection bias may have influenced our results. We found that students who remained in the analysis were on average older and more likely to have asthma; however, sensitivity analyses replicating our models using the full sample did not yield different results (Table S1).

The present study expands upon the distance–gradient method of exposure analysis, a simple and frequently used technique in environmental health research, using information exclusively obtained from school district records [57]. A principal limitation of this study was a lack of direct air quality measures as well as in-home exposures, such as smoking, cleaning products, pets, molds, etc. [27]. As demonstrated by Bergstra et al., the addition of this information, together with distance-based analysis, can allow for a significantly more robust and conclusive analysis about the effects of industry on pediatric respiratory health [27]. However, the distance–gradient method of exposure analysis has the important advantage of being accessible to historically under-resourced communities that may not be able to afford extensive data collection devices or field campaigns. This approach allows us to focus on multiple factors (e.g., air pollution, lack of green space, noise) combined with zoning effects, in contrast to a single measure of an exposure variable. In addition, the results on the zoning effects may have direct implications for zoning policy changes in the city.

Future studies could build upon our results using similar cost-effective research approaches, e.g., testing distance–gradient exposure models on school districts with similar baseline characteristics, to examine whether industrial zone proximity can successfully predict asthma or other health risks even in the absence of point source information. Such models could be expanded to include the effect of possible asthma-protective factors, such as green space [55]. Future studies could also leverage the longitudinal school records to observe changes in asthma incidence and severity over time. Resource-intensive approaches, including the deployment of high-resolution air monitoring technology, the measurement of additional physiologic outcomes through pulmonary function testing, and expanded surveys to measure in-home exposures, could subsequently be employed to elucidate the specific modifiable risk factors that mediate the effects of industrial zone proximity on the observed pediatric health risks.

## 5. Conclusions

Our findings suggest that industrial zone proximity is associated with increased pediatric asthma in a predominantly poor and Latinx community in Southern California. We utilized a simplified approach to environmental risk factor identification, in which industrial zones are seen not just as potential sources of air pollution, but as dynamic entities with effects on population health that can be quantified and managed, even in the absence of sophisticated and expensive sampling equipment. At a local level, these results provide important context for the intersection of city zoning policy and children’s asthma health, which may be immediately valuable to local environmental justice groups, school district health staff, and city policymakers. Future studies could examine the possible protective effects of green space and/or other recreational areas on asthma and weight-related outcomes. Lastly, the use of school district records was possible through our collaborations with the local school district and highlights how schools/communities and universities can collaborate to study the intersection between pediatric health, environmental exposures, and academic outcomes.

## Figures and Tables

**Table 1:** Sample characteristics.

	Overall *n* (%)/ Mean ± SD	Asthma *n* (%)/ Mean ± SD	Non-Asthma *n* (%)/ Mean ± SD	Asthma vs. Non-Asthma
				*p*-Value ^1^
Total	39,974	2747 (6.9)	37,227 (93.1)	
Demographics				
Age in years	12.2 ± 3.6	12.5 ± 3.4	12.2 ± 3.6	<0.01
Male	20,198 (50.5)	1589 (57.8)	18,609 (50.0)	<0.01
Hispanic	32,226 (80.6)	2179 (79.3)	30,047 (80.7)	0.08
Parents’ education (HS diploma)	19,052 (47.7)	1159 (42.2)	17,893 (48.1)	<0.01
Free/reduced lunch	35,257 (88.2)	2348 (85.5)	32,909 (88.4)	<0.01
Fitness				
Overweight/obese	4910 (50.7)	451 (58.4)	4459 (50)	<0.01
Failed aerobic test	4205 (44.0)	378 (49.8)	3827 (43.5)	<0.01
Academic				
Failed SB math test	16,200 (74.0)	1135 (72.4)	15,065 (74.1)	0.15
Failed SB English test	14,743 (67.4)	1061 (67.5)	13,682 (67.3)	0.89
Health/medical absences	3.3 ± 4.7	4.9 ± 6.6	3.2 ± 4.5	<0.01
Total absences	5 ± 7.3	6.8 ± 9.1	4.9 ± 7.1	<0.01
Distance measures				
Distance to industrial zone (km)	0.9 ± 0.8	0.8 ± 0.8	0.9 ± 0.8	0.08
Distance to industrial zone (categorical)				<0.01
Closest (<0.5 km)	13,323 (33.3)	803 (29.3)	12,519 (33.6)	
Middle (0.5–1.0 km)	13,340 (33.4)	974 (35.5)	12,366 (33.2)	
Farthest (>1.0 km)	13,311 (33.3)	969 (35.3)	12,342 (33.2)	
Distance to freeway (km)	2.22 ± 1.06	2.16 ± 1.06	2.22 ± 1.06	<0.01
Distance to freeway (categorical)				0.09
Closest (<1.5 km)	13,369 (33.4)	881 (32.1)	12,488 (33.5)	
Middle (1.5–3.0 km)	13,265 (33.2)	899 (32.7)	12,366 (33.2)	
Farthest (>3.0 km)	13,340 (33.4)	967 (35.2)	12,373 (33.2)	

^1^ *p*-Value comparing asthma vs. non-asthma groups corresponds to *t*-test for continuous variables and chi-squared test for categorical variables.

**Table 2:** Regression models of asthma and academic outcomes by distance to industrial zones and freeways.

Outcome	Prevalence	Distance to Industrial Zone (vs. *Farthest Tertile*, >1.0 km)				Distance to Freeways (vs. *Farthest Tertile*, >3.0 km)			
		Closest (<0.5 km)		Middle (0.5–1.0 km)		Closest (<1.5 km)		Middle (1.5–3.0 km)	
	*n* (%)	aOR (95% CI)	*p*-Value	aOR (95% CI)	*p*-Value	aOR (95% CI)	*p*-Value	aOR (95% CI)	*p*-Value
Asthma diagnosis among all students (*n* = 39,974) *									
	2747/39,974 (6.9%)	**1.21 (1.09, 1.34)**	**<0.001**	**1.21 (1.10, 1.34)**	**<0.001**	**1.18 (1.03, 1.35)**	**0.014**	1.03 (0.92, 1.16)	0.555
Overweight, fitness, and academic outcomes among students with asthma *									
Overweight or obese	451/772 (58.4%)	1.20 (0.80, 1.80)	0.371	**1.47 (1.00, 2.15)**	**0.047**	0.64 (0.38, 1.07)	0.091	0.87 (0.57, 1.32)	0.513
Failed aerobic fitness test	378/759 (49.8%)	1.29 (0.86, 1.92)	0.219	1.21 (0.83, 1.76)	0.33	1.17 (0.70, 1.96)	0.55	0.82 (0.55, 1.25)	0.358
Failed SB math test	1135/1567 (72.4%)	1.00 (0.74, 1.35)	0.995	1.26 (0.94, 1.68)	0.124	0.81 (0.54, 1.20)	0.288	0.84 (0.60, 1.16)	0.286
Failed SB ELA test	1061/1571 (67.5%)	1.05 (0.78, 1.40)	0.759	1.17 (0.89, 1.55)	0.267	0.68 (0.47, 1.00)	0.051	0.79 (0.57, 1.09)	0.151
Attendance among students with asthma *									
	***n* (Median, IQR)**	**Coef** **(95% CI)**	***p*-value**	**Coef** **(95% CI)**	***p*-Value**	**Coef** **(95% CI)**	***p*-Value**	**Coef** **(95% CI)**	***p*-Value**
Total absences	2747 (4, 1–9)	−0.09 (−1.02, 0.84)	0.845	−0.22 (−1.10, 0.66)	0.627	−0.60 (−1.80, 0.60)	0.326	0.33 (−0.66, 1.32)	0.515
Health-related absences	2747 (3, 1–7)	−0.18 (−0.85, 0.50)	0.608	−0.15 (−0.79, 0.48)	0.633	−0.11 (−0.97, 0.76)	0.808	0.38 (−0.34, 1.10)	0.301

* Outcomes were modeled using logistic regression or linear regression (total and health-related absences only). Each model includes regression terms for composite distance to industrial zone or freeway (categorical) as the main independent variable, as well as gender, age, race/ethnicity, free or reduced lunch qualification status, and a dummy variable for zip code. Sample sizes differ due to incomplete data and/or test eligibility (some tests administered only to certain grades).

## Data Availability

Not applicable.

## Supplementary Materials

The following supporting information can be downloaded at: https://www.mdpi.com/article/10.3390/ijerph19084820/s1, Table S1: Sensitivity analysis among full sample.
