# Supplementary material for: Breathing Room: Industrial Zoning and Asthma Incidence Using School District Health Records in the City of Santa Ana, California
Source: Int J Environ Res Public Health. 2022 Apr 15;19(8):4820. doi: 10.3390/ijerph19084820 (PMC9032322; doi:10.3390/ijerph19084820)
Supplement: Supplementary file 1 [file ijerph-19-04820-s001.zip › ijerph-1644727-SI.pdf]

Table S1. Sensitivity analysis among full sample.

Outcome	Prevalence	Distance to Industrial Zone (Tertiles)				Distance to Freeways (Tertiles)			
		**Closest (<0.5 km)**	***p*-Value**	**Middle (0.5–1.0 km)**	***p*-Value**	**Closest (<1.5 km)**	***p*-Value**	**Middle (1.5–3.0 km)**	***p*-Value**
	***n* (%)**	**aOR (95% CI)**		**aOR (95% CI)**		**aOR (95% CI)**		**aOR (95% CI)**	
All students (n = 44,641)									
**Asthma diagnosis**	2987/44,641 (6.7%)	**1.21 (1.09, 1.34)**	**<0.001**	**1.20 (1.09, 1.33)**	**<0.001**	**1.17 (1.03, 1.34)**	**0.019**	1.03 (0.92, 1.15)	0.637
Presumed asthma diagnosis	3173/44,641 (7.1%)	**1.16 (1.05, 1.28)**	**0.003**	**1.19 (1.08, 1.31)**	**<0.001**	1.13 (0.99, 1.28)	0.073	1.02 (0.92, 1.14)	0.699
Among those with asthma diagnosis*									
Overweight or obese	480/823 (58.3%)	1.19 (0.80, 1.78)	0.389	1.46 (1.00, 2.13)	0.052	0.64 (0.38, 1.07)	0.089	0.87 (0.57, 1.33)	0.52
Failed aerobic fitness test	404/809 (49.9%)	1.28 (0.86, 1.90)	0.231	1.19 (0.82, 1.73)	0.361	1.17 (0.70, 1.95)	0.556	0.83 (0.55, 1.25)	0.369
Failed SB math test	1215/1667 (72.8%)	1.00 (0.74, 1.36)	0.992	1.25 (0.94, 1.68)	0.13	0.81 (0.54, 1.20)	0.289	0.83 (0.60, 1.17)	0.295
Failed SB ELA test	1137/1,672 (68.0%)	1.05 (0.78, 1.41)	0.747	1.17 (0.88, 1.54)	0.277	0.68 (0.47, 1.00)	0.051	0.79 (0.57, 1.09)	0.157
	***n* (Median, IQR)**	**Coef (95% CI)**	***p*-Value**	**Coef (95% CI)**	***p*-Value**	**Coef (95% CI)**	***p*-Value**	**Coef (95% CI)**	***p*-Value**
Among those with asthma diagnosis*									
Total absences	2,757 (3, 1–7)	−0.18 (−0.85, 0.49)	0.6	−0.15 (−0.78, 0.49)	0.654	−0.11 (−0.98, 0.75)	0.798	0.36 (−0.36, 1.08)	0.325
Health-related absences	2.757 (4, 1–9)	−0.14 (−1.07, 0.79)	0.772	−0.23 (−1.11, 0.65)	0.604	−0.61 (−1.81, 0.59)	0.317	0.30 (−0.69, 1.30)	0.548

* Sample sizes differ due to incomplete data and/or test eligibility (some tests administered only to certain grades).
